# A call for high-intensity lipid-lowering treatment of ASCVD patients diagnosed by coronary computed tomography angiography: lessons from the multi-center LOCATE study

**DOI:** 10.1007/s00392-025-02604-9

**Published:** 2025-02-11

**Authors:** Franz Haertel, Ulf Teichgräber, P. Christian Schulze, Oliver Weingärtner

**Affiliations:** 1 Department of Internal Medicine I, Cardiology, University Hospital Jena, Am Klinikum 1, 07747 Jena, Germany; 2 Department of Radiology, University Hospital Jena, Am Klinikum 1, 07747 Jena, Germany

Sirs:

A recent analysis by the Federal Institute for Population Research of Germany (Bundesinstitut für Bevölkerungsforschung (BIB)), led by Jasilionis and colleagues, paints a concerning picture of life expectancy trends in Germany.

Using comparative analyses of life expectancy derived from life tables obtained from the Human Mortality Database, along with age and cause-specific decomposition, the authors provided robust evidence that Germany is currently lagging behind other developed countries in life expectancy gains [1].

Germany ranks last among high-income European Union countries, with a life expectancy at least 2 years lower, primarily due to underperformance in cardiovascular disease prevention.

Contrasting this fact, Germany has the highest per capita expenditures on cardiovascular diseases within the European Union [2]. With more than 900 Euros spent per person annually, Germany is the unchallenged leader in cardiovascular disease expenditures in Europe [2].

The classical risk factors for atherosclerotic cardiovascular diseases (ASCVD) are highly modifiable and account for nearly 70% of all cardiovascular diseases in high-income countries [3]. In other words, ASCVD are highly preventable.

These findings have prompted a new initiative by the Federal Ministry of Health of Germany (Bundesministerium für Gesundheit (BMG)) called the “Gesetz zur Stärkung der Herzgesundheit” (“Gesundes—Herz-Gesetz” = “Healthy Heart Initiative”) [4].

A key component of this draft legislation, in addition to screening for familial hypercholesterolemia in early childhood, is to enhance the ability to prescribe statins for patients at risk. With this new legislation, it is estimated that around 2 million more patients in Germany could become eligible for statin treatment and thus included into a preventive, risk factor modifying treatment plan.

Furthermore, the Federal Joint Committee (Gemeinsame Bundesausschuss (G-BA)) has recently endorsed the use of cardiac computed tomography angiography (CCTA) as a screening tool for ASCVD [5]. This decision is expected to significantly increase the use of CCTA, leading to the identification of hundreds of thousands of patients with newly diagnosed atherosclerotic lesions. The pressing question that will inevitably arise is: “How do we treat these patients?”.

The differing treatment approaches (“fire-and-forget” vs. “treat-to-target”) and low-density-lipoprotein cholesterol (LDL-C) targets (< 70 mg/dl vs. < 55 mg/dl) between the National Treatment Guideline (Nationale Versorgungsleitlinie) endorsed by the German Society of General Practice/Family Medicine (Deutsche Gesellschaft für Allgemeinmedizin und Familienmedizin e.V. (DEGAM)) and the European Society of Cardiology / European Atherosclerosis Society (ESC/EAS) Dyslipidemia Guideline endorsed by the German Cardiac Society (Deutsche Gesellschaft für Kardiologie (DGK)) have sparked a controversial debate in Germany [6, 7]. The results of LOCATE offer new insights into this controversy and provide valuable answers to this critical issue.

In the current issue of *Clinical Research in Cardiology*, Weichsel L. and colleagues present the results of the LOCATE study. This multi-center observational study evaluated the effects of varying intensities of lipid-lowering medications on the total volumes of calcified and non-calcified plaques in 216 patients undergoing cardiac computed tomography angiography (CCTA).

Progression of total and non-calcified plaques was significantly reduced in patients on moderate- to high-intensity lipid-lowering treatment compared to those on no or low-intensity treatment after a mean follow-up time of more than 2 years. Remarkably, patients on high-intensity statins or more intensive treatments, including PCSK9 inhibitors, even showed a reversal of non-calcified plaques. The authors concluded that these findings establish a mechanistic link between high-intensity lipid-lowering therapy, more effective LDL-C reductions, and the stabilizing effects on atherosclerotic lesions, potentially leading to improved cardiac outcomes.

The key takeaway from the study is that high-intensity lipid-lowering treatment can halt or even reverse plaque progression, whereas moderate-intensity treatment may not achieve this effect. The authors recommend considering higher doses of potent statins, such as atorvastatin > 40 mg or rosuvastatin > 20 mg, or combined lipid-lowering therapy in high-risk patients to potentially reduce cardiovascular events. These findings align with recent reports on patients with acute myocardial infarction [8]. The PACMAN-AMI study demonstrated that intensive lipid-lowering therapy, or “delipidation,” resulted in a higher percentage of lesions exhibiting triple regression-reduced atheroma volume, decreased lipid core burden, and increased fibrous cap thickness-on serial multimodality intracoronary imaging [9]. These improvements were associated with better clinical outcomes during follow-up after the initial event.

Regrettably, real-world data on LDL-C target attainment in high-risk patients in Germany reveal a significant treatment gap. The LIPIDSNAPSHOT study—a collaborative research project by the Center for Health Services Research of the German Cardiac Society (DGK-ZfKVF), the Federal Association of Office-Based Cardiologists (BNK), and the German Society of Lipidology (DGFL)-Lipid-Liga—evaluated differences in lipid-lowering treatment (LLT) and LDL-C target attainment in ASCVD patients treated by office-based cardiologists (OBCs) and general practitioners (GPs) in Germany in 2023 [10]. In this extensive analysis, which included nearly 84.000 high-risk patients, the average LDL-C level was 75 mg/dl in those treated by cardiologists and 96 mg/dl in those treated by GPs. Notably, age-related analysis showed that among patients under 50 treated by GPs, half did not receive any lipid-lowering medication. Given that early-onset ASCVD patients represent the highest-risk group, this study starkly highlights the significant undertreatment of high-risk patients in Germany.

On the other hand, the prospective cohort study “Jena auf Ziel” has shown that achieving LDL-C targets is both feasible and well-tolerated in high-risk patients [11]. 80% of high-risk patients achieved the ESC/EAS LDL-C targets with atorvastatin 80 mg and ezetimibe 10 mg. For those who required further reduction, the addition of bempedoic acid or PCSK9 inhibitors ensured that all patients reached their LDL-C goals (≤ 1.4 mmol/L).

Moreover, imaging studies utilizing computed tomography or carotid ultrasound have demonstrated that visualization of plaques by vascular imaging improves prescriptions by physicians and patient adherence. [12–14].

The authors of this editorial suggest that the discrepancies in treatment outcomes between patients managed by office-based cardiologists (OBCs) and general practitioners (GPs) are primarily due to the differing recommendations in their respective guidelines. The study by Weichsel and colleagues offers compelling new insights into this issue.

**Fig. 1:**
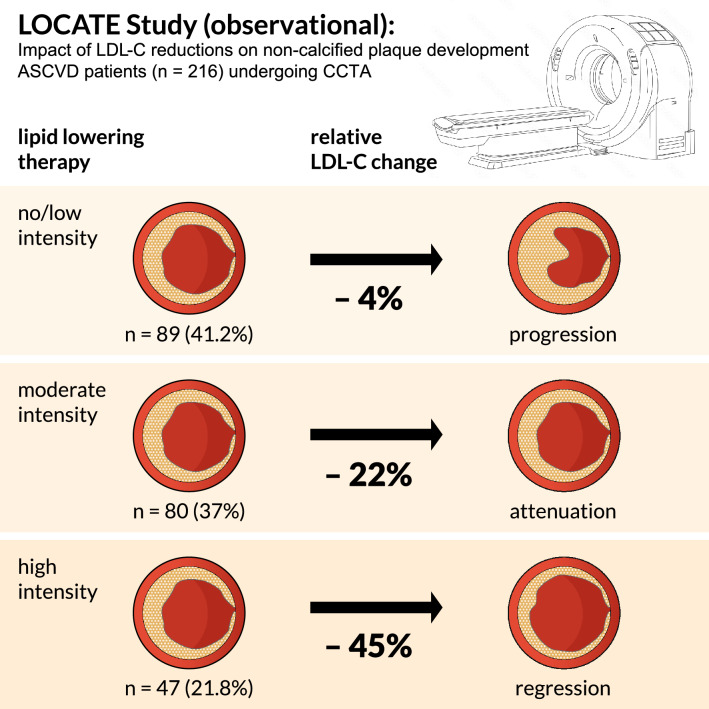
Impact of lipid-lowering treatment intensity on atherosclerotic lesion progression in ASCVD patients: Insights from CCTA assessments

First, LOCATE highlights the value of CCTA as a screening tool for quantifying and monitoring atherosclerotic lesions. More importantly, LOCATE is the first study to demonstrate that, in ASCVD patients diagnosed by CCTA, high-intensity lipid-lowering therapy is superior to moderate- or low-intensity treatments. It not only reverses atherosclerotic plaques but also potentially improves cardiovascular outcomes. These findings underscore the superiority of the ESC/EAS recommendation for target-based, intensive lipid-lowering treatment over the less intensive approach currently advocated by the German Society of General Practice/Family Medicine (Deutsche Gesellschaft für Allgemeinmedizin und Familienmedizin e.V. (DEGAM)). Lastly, LOCATE’s results will strengthen the initiative by the Federal Ministry of Health of Germany (Bundesministerium für Gesundheit (BMG)) to enhance the early prescription of statins for ASCVD patients in Germany, aiming to further reduce cardiovascular risk and long-term healthcare costs associated with major adverse cardiac events (MACEs). Nevertheless, LOCATE has to be considered as a pilot study that has to be confirmed on the basis of larger patient cohorts, a Corelab ajudicated evaluation and, above all, a potential clinical benefit (Fig. 1).
